# Evaluation of malaria surveillance system in Benue State, Nigeria

**DOI:** 10.1186/s12936-022-04367-4

**Published:** 2022-11-23

**Authors:** Peter Okpeh Amede, Chukwuma David Umeokonkwo, Susan Abege, Joseph Akawe, Jeh Derek, Elizabeth Adedire, Muhammad Shakir Balogun

**Affiliations:** 1 Nigeria Field Epidemiology and Laboratory Training Programme, Abuja, Nigeria; 2 Department of Community Medicine, Alex Ekwueme Federal University Teaching Hospital, Abakaliki, Ebonyi State Nigeria; 3 Benue State Ministry of Health, Makurdi, Nigeria; 4 African Field Epidemiology Network, Abuja, Nigeria

**Keywords:** Malaria, Surveillance system, Attributes, Benue State, Nigeria

## Abstract

**Background:**

Malaria is a priority global health disease with high morbidity and mortality especially among children under-five and pregnant women. Malaria elimination requires an effective surveillance system. The malaria surveillance system in Benue State was evaluated to assess its attributes and performance in line with set objectives.

**Methods:**

The updated United States Centers for Disease Control and Prevention guideline for evaluating surveillance systems was used. The surveillance system’s key attributes was quantitatively and qualitatively assessed. Semi-structured questionnaires were administered to all Local Government Area (LGA) Roll Back Malaria (RBM) focal persons and five key informants were interviewed at the State level. The Benue State District Health Information System-2 (DHIS-2) malaria data and monthly summary forms were reviewed from January 2015 to December 2019.

**Results:**

A total of 46 RBM focal persons and 5 key-informants participated. About 56.9% were males, the mean-age 43.8 (SD ± 9.3) years and 32 (62.8%) had ≥ 20-year experience on malaria surveillance with mean-year-experience 20.8 (SD ± 7.8) years. All 46 (100%) RBMs understood the case definition; 43 (93.5%) found it easy-to-fill the standardized data tools and understood the data flow channels. The malaria surveillance system in Benue is simple, acceptable and useful to all stakeholders, 36 (70.6%) found switching from the paper-based to the electronic-data tools with ease and 45 (88.2%) stated that analysed data were used for decision-making. Data flow from LGA to State is clearly defined, however majority of the data is collected from public health facilities through the DHIS-2 Platform. The overall timeliness and completeness of reporting was 76.5% and 95.7%, respectively, which were below the ≥ 80% and 100% targets, respectively.

**Conclusions:**

The malaria surveillance system in Benue State is simple, useful, acceptable, and flexible, but it is not representative and timely. Public–private and public-public-partnerships should be strengthened to encourage reporting from both private and tertiary health facilities and improve representativeness, and frequent feedback to improve reporting timeliness.

## Background

Malaria is still a major public health concern and endemic in Nigeria. In 2019, the World Health Organization (WHO) established that, 227 million cases and 409,000 malaria deaths occurred globally and Africa accounted for 94% (213 million cases) of the cases and 94% (386,000 deaths) of the deaths [1]. Although malaria is preventable, treatable and curable, Nigeria, accounted for 27% (61.8 million cases) of the global burden and 23% (94, 070 deaths) of global malaria deaths in 2019 [1]. Nigeria, is among the six countries that accounted for 51% of all malaria cases globally in 2019 [1]. Pregnant women and children under the age of five are the most vulnerable populations in Nigeria. Malaria is responsible for 60% of all outpatient visits and 30% of all admissions in Nigeria. It is responsible for about 11% of maternal fatalities and 30% of child mortality among children under the age of five [2]. Malaria further weakens the country's already frail health system and imposes a tremendous socioeconomic burden, depressing the country's Gross Domestic Product (GDP) by 40% yearly and costing the country about 480 billion naira in direct and indirect medical costs [3]. The evaluation of the malaria surveillance system in Benue State is critical for tracking progress toward elimination.

The continual, systematic collection, analysis, interpretation, and dissemination of data about a health-related event for use in public health action to reduce morbidity and mortality and promote health is known as public health surveillance [4]. The evaluation of public health surveillance systems promotes the most efficient and effective use of health resources by ensuring that only the important problems are under surveillance and that the systems run smoothly. It is critical to assess the malaria surveillance system to ensure that high-quality data is used to provide information for planning, focusing interventions, and monitoring malaria programmes [5].

Surveillance is the cornerstone of disease prevention and control in settings of any level of transmission. Surveillance is crucial to malaria elimination programmes because it provides the data needed to target interventions and track their effectiveness [6, 7]. The National Malaria Elimination Programme (NMEP) is one of the national malaria response programmes that key into the Global Technical Strategy (GTS) for malaria 2016–2030 [8]. The goal of this strategy is to minimize the burden of malaria, eliminate the disease, and prevent its re-emergence. The NMEP aims to reduce malaria burden to pre-elimination levels and eliminate malaria-related death. In settings in which transmission remains relatively high and the aim of national programmes is to reduce the burdens of morbidity and mortality, malaria surveillance is often integrated into broader routine health information systems to provide data for overall trend analysis, stratification and planning of resource allocation [8].

Malaria programmes in Benue State have failed to control the incidence of the disease, and the state's malaria burden remains high. Evaluation of the malaria surveillance system in Benue State was conducted in order to assess its attributes and performance in relation to defined objectives. The findings of the study can be applied to targeted intervention, public health action, and planning.

## Methods

### Study area

Benue State lies within the lower river Benue trough located in the North-central region of Nigeria. Its geographic coordinates are longitude 7^°^ 47^**′**^ and 10^**°**^ 0^**′**^ East, Latitude 6^**°**^ 25^**′**^ and 8^**°**^ 8^**′**^ North. It shares boundaries with five other States namely Nassarawa to the north, Taraba to the east, Cross River to the south, Enugu to the south-west and Kogi to the west. The State shares a common boundary with the Republic of Cameroon on the south-east. Benue State has a population of 4,780,389 people (NPC: 2006) and occupies a landmass of 34,059 square kilometres. Benue State lies within the AW climate and experiences two distinct seasons, the wet/rainy season and the dry/harmattan season. The rainy season lasts from April to October with annual rainfall in the range of 100-200 mm and the dry season begins in November and ends in March with temperature fluctuates between 21 and 37 °C in the year [9]. Malaria is endemic in Benue State, and its transmission peaks between May and September, reflecting the period of high mosquito density sustained by the climatic condition such as temperature an important determinant of growth and development of immature mosquitoes [10–12]. There are 23 Local Government Areas (LGAs) in Benue State with 276 political wards and 3 senatorial districts.

Benue State has 1408 Health Facilities (HF) compressing of 862 Primary Health Care (PHC) facilities, 24 Secondary health care facilities, 2 tertiary health care facilities and 520 registered private health facilities. One thousand one hundred and forty-two out of the 1408 HF (81.0%) in the State have harmonized Health Management Information System (HMIS) data capturing tools.

The Benue State Malaria Elimination Programme (BSMEP) officers are responsible for malaria control at the State level while the LGA Roll Back Malaria (RBM) Focal Persons are responsible for malaria control at their respective LGA. Treatment of malaria in designated HF in the State is free for adults and children.

### Malaria surveillance system operation in Benue State

The surveillance system consists of operators at the State and LGA levels. For the system's effective and efficient operation, these operators collect, collate, and analyse data. The state and LGA malaria programme managers, who provide technical assistance and training, and the LGA monitoring and evaluation (M&E) officers, who actually collect and enter the data into the DHIS-2 platform, are among the key stakeholders.

**Fig. 1:**
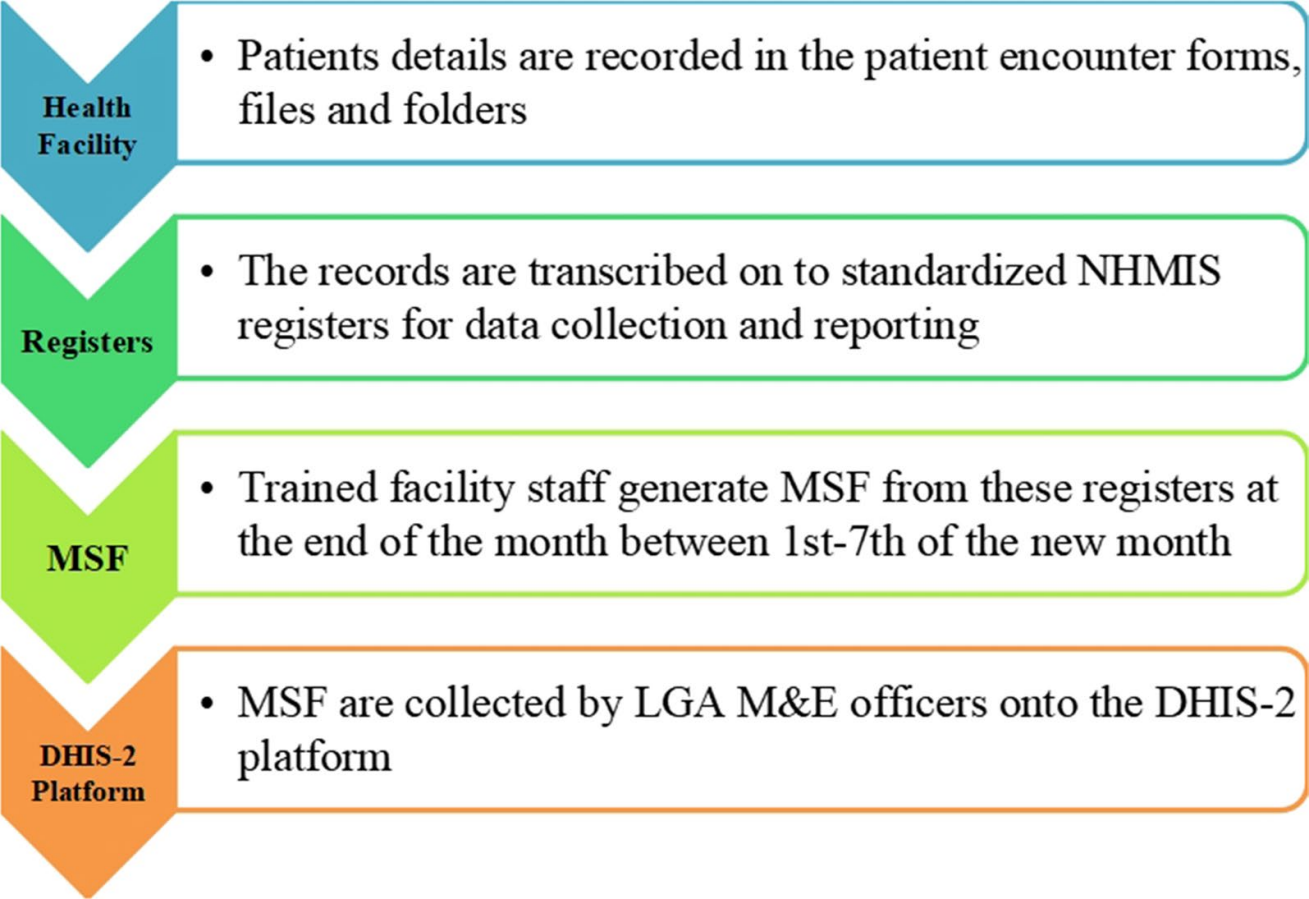
Data flow from Health facility to the LGA DHIS-2 Platform in Benue State

Malaria surveillance in Benue State is passive and ongoing throughout the year, involving the collecting and entry of malaria data into the National Health Management Information System (NHMIS)-Monthly Summary Form (MSF) by designated officers at health facilities (NHMIS-MSF) (Fig. 1).

**Fig. 2:**
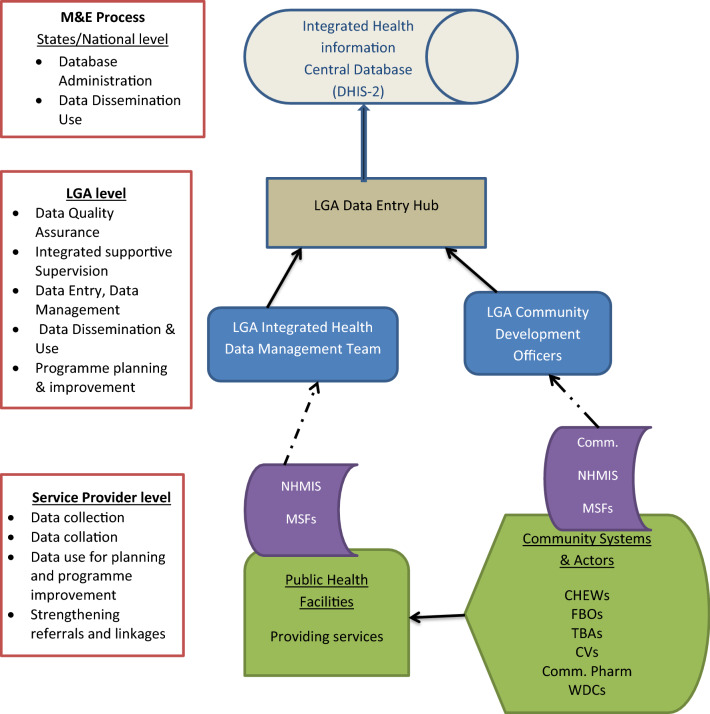
Operation of Benue State malaria surveillance system

The LGA M&E officers collect these forms from the health facilities, compile the information, and enter it into the DHIS-2 database. Designated officers at the national and state levels have access to these data for decision-making and feedback. The malaria data flow in Benue state was adopted from the National Malaria Elimination Programme’s standard operating procedure for data management in its revised edition [13]. (Fig. 2).

### Study design

Descriptive cross-sectional study was conducted, which consists of retrospective review and analysis of District Health Information System-2 (DHIS-2) malaria-data from January 2015 to December, 2019 and as well as a survey among Roll Back Malaria focal persons (RBMs) at the 23 LGAs and key informants at the State level. RBMs are health care workers that coordinate malaria activities at the LGAs.

### Data collection

The evaluation was conducted in January, 2020 using the United States Centers for Disease Control and Prevention (CDC), 2001 updated guidelines for evaluation of public health surveillance systems [4]. Mixed method of data collection was used to obtain information from 46 Roll Back Malaria (RBM) focal persons selected purposively from 23 LGAs in Benue State, using standardized semi-structured self-administered questionnaires (Appendix A) and Key Informant Interview guide, adopted from a previous surveillance evaluation [14] (Appendix B); were conducted among five-key-informants which comprised the Benue State Malaria Programme manager, the State malaria programme Monitoring and Evaluation (M&E) officer, State epidemiologist, State Disease Surveillance and Notification Officer (DSNO) and the State M&E officer to obtain their inputs in describing the system, assessing key attributes of the system and to ensure that findings from the evaluation will be executed and reviewed District Health Information System-2 (DHIS-2) malaria data, a web-based platform from January 2015- December, 2019. Socio-demographic characteristics, years of experience, laboratory and epidemiologic variables and attributes of the surveillance system such as simplicity, acceptability, usefulness, flexibility, representativeness, timeliness and reporting rate was collected.

### Data analysis

The surveillance system’s key attributes was evaluated quantitatively and qualitatively, and then the findings was compared to the standards in the US Centers for Disease Control and Prevention’s (CDC) 2001 updated guidelines for evaluating public health surveillance systems. The quantitative data from the survey and abstracted data from the Benue State DHIS-2 platform were analysed using Epi-info 7.0 and Microsoft Excel 2016. Frequencies, proportions, means, and standard deviation were used to summarize the data, which was then displayed in charts.

Malaria positivity rate was measured as the number of positive tests (RDT and/or microscopy) in a given period as a proportion of the total number of tests (positive tests plus negative tests) in same period. The testing rate is the ratio of febrile cases tested in a given period to the total febrile cases reported in a given period. The “number of monthly reports received from health facilities within a given time period of on or before the 5th day of the new month, as a proportion of expected total number of health facility reports” was used to determine the timeliness of reporting. For each year, this was computed. The “number of monthly reports received from health facilities as a fraction of expected total number of health facility reports” was used to calculate the reporting rate/completeness of reporting for each year. Non-reporting health facilities were excluded from the calculation. The KII were evaluated thematically, and similar responses were grouped together. The qualitative assessment was carried out by measuring key indicators such as the system's adaptability to changes, data quality, and the programme's funding sources, as well as training and supportive supervision.

## Results

### Socio-demographic characteristics

A total of 46 Roll Back malaria Managers (RBMs) and 5 key informants participated in the study. The mean and standard deviation of their age was 43.8 ± 9.3 years. A greater proportion 56.9% (29/51) were males, 68.6% (35/51) were aged ≥ 40 years, and 32/51 (62.7%) had ≥ 20-year experience on malaria surveillance with mean-year-experience 20.8 (SD ± 7.8) years. Twenty-eight (60.9%) of the RBMs were community health officers, 11 (23.9%) environmental officers and 7 (15.2%) nurses.

During the 5-year review period, 1,470,523 cases of fever were abstracted from the Benue State DHIS-2, with 899,480 (61.2%) being laboratory-confirmed to be malaria. A majority of the confirmed cases were uncomplicated 880,811 (97.9%), while 18,669 (2.1%) were severe malaria. A total of 123,763 of the 899,480 reported malaria cases (13.8%) were malaria in pregnancy. Of the laboratory confirmed cases, 865,389 (96.2%) were RDT-confirmed and 34,091 (3.8%) were microscopy confirmed as depicted in Table 1.

**Table 1:** Clinical and laboratory characteristics of fever cases; January 2015-December 2019

Variable	Year					Total
	2015	2016	2017	2018	2019	
Fever	273085	276725	314063	383179	223471	1470523
Clinical malaria	66160	36124	16436	29840	19836	168396
Laboratory confirmed	161379	167207	187143	247997	135754	899480
mRDT	153976	158995	178205	241959	132254	865389
Microscopy	7403	8212	8938	6038	3500	34091
Uncomplicated malaria	156212	163145	185580	245204	130670	880811
Severe malaria	6107	2959	1702	5578	2323	18669

### Benue State malaria surveillance system attributes

#### Usefulness

**Fig. 3:**
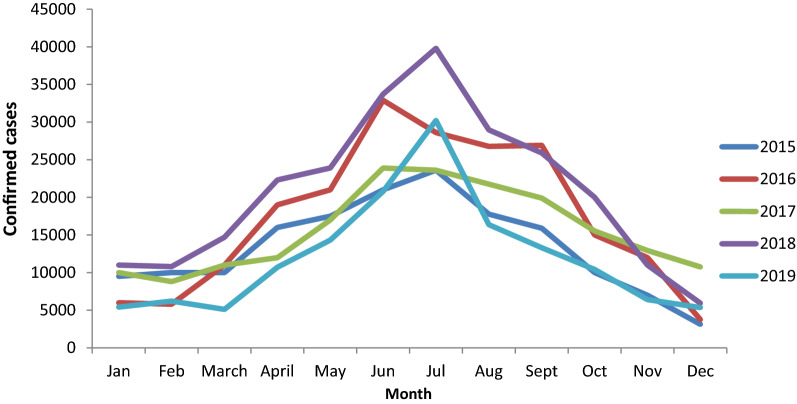
Trend of confirmed malaria cases in Benue State, January 2015-December 2019

**Fig. 4:**
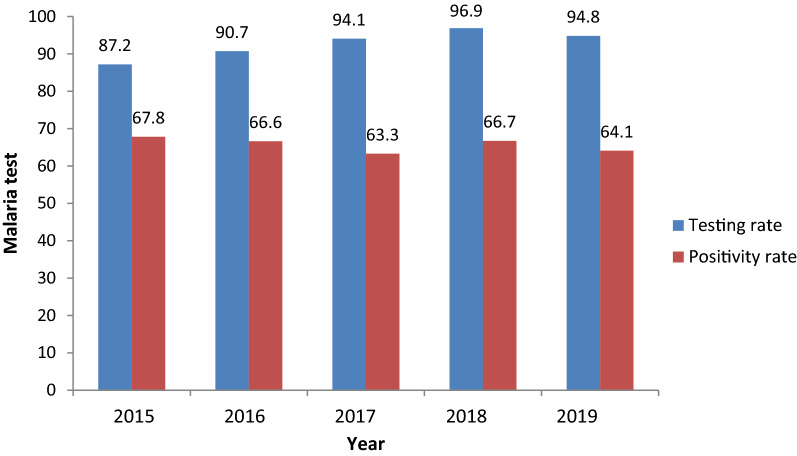
Trend of malaria test among febrile cases in Benue State, 2015–2019

The malaria surveillance system in Benue State was established to detect incidence of malaria quickly and to alert the system if there was an unusual increase in malaria cases as stated by the key stakeholders and all the RBMs said the system detects increase in malaria cases over the years. Weekly mean of malaria cases in each LGA is used as the alert trigger as there is no set threshold for alert. If the mean of malaria cases of a certain week is clearly in excess of the corresponding week mean of the previous year, the system is alerted of a possible outbreak and the LGA disease surveillance and notification officer will institute a quick investigation to verify the possibility of an outbreak. Over the years, the approach has proven to be effective in detecting cases. Over the course of the 5 years, 1,470,523 malaria cases were detected. During the study period, malaria cases were more prevalent from April to September each year, with peaks in June and July. The trend of confirmed malaria cases during the study period was shown in Fig. 3. The testing rates by RDT and microscopy increased steadily over the period and this was compared with positivity rate (Fig. 4). All stakeholders reported that findings from the system were used for decision-making.

#### Simplicity

Simplicity relates to the system’s structure as well as the ease with which it can be used. All 46 (100%) RBM focus persons and the 5 (100%) key state stakeholders described the system as simple, and the case definitions were well understood. The data flow channel was well-defined, precise, and followed. Forty-three (93.5%) of the RBMs said the paper-based forms (National Health Management Information System-Monthly Summary Forms) used to collect data, are simple to complete out. The case definitions and screening test process were both basic, and the tools were simple to fill out, according to all five (100%) state stakeholders. The respondents unanimously agreed that entering data into the DHIS-2 was uncomplicated.

#### Acceptability

Individuals and organizations’ willingness to participate in the monitoring system is referred to as acceptability. All 46 respondents (100%) said they will continue to use the surveillance system. Thirty-four (73.9%) of respondents believe the system recognizes their efforts in doing their jobs well and that their suggestions for enhancing the system are implemented.

#### Flexibility

The ability of a system to adjust to changing needs is known as flexibility. The system adapted well to the newly revised national standard operating procedure for malaria surveillance, monitoring, and evaluation because it accommodated changes in the national diagnosis and treatment guideline of 2011 and revised in 2015, from presumptive clinical diagnosis and monotherapy treatment to the current policy of parasitological diagnosis by RDT or microscopy and ACT treatment. Forty of the 46 (87.0%) RBMs indicated that changes in data tools and reporting sources were accommodated with negligible impact on effort and 13.0% said the changes were accommodated poorly due to inadequate staff to accomplish tasks on time and more training was needed. Thirty-eight of the 46 (82.6%) RBMs said the training and occasional supportive supervision helped them make a smooth transition. The system adapted well to modifications in data capture tools and treatment guidelines, according to all five major stakeholders, and the main resource used to implement these changes was human.

#### Representativeness

Data for the period evaluated were from primary and secondary public health facilities and from 25 of the 520 private health facilities, and none from the 2 tertiary health facilities in the state therefore the surveillance system in Benue State was not representative of all the health facilities. The main challenge in the system, according to key stakeholders, is a lack of data from majority of the private health facilities and none from the tertiary health facilities, as well as irregular reporting from some secondary health facilities due to staffing shortages.

#### Completeness of reporting/reporting rate

Completeness of reporting is the total number of LGAs that reported for the month in a year irrespective of time of reporting. The annual target for reporting rate is 100% and was achieved in the last 3 years (2017–2019) of the evaluation period.

#### Timeliness of reporting

**Fig. 5:**
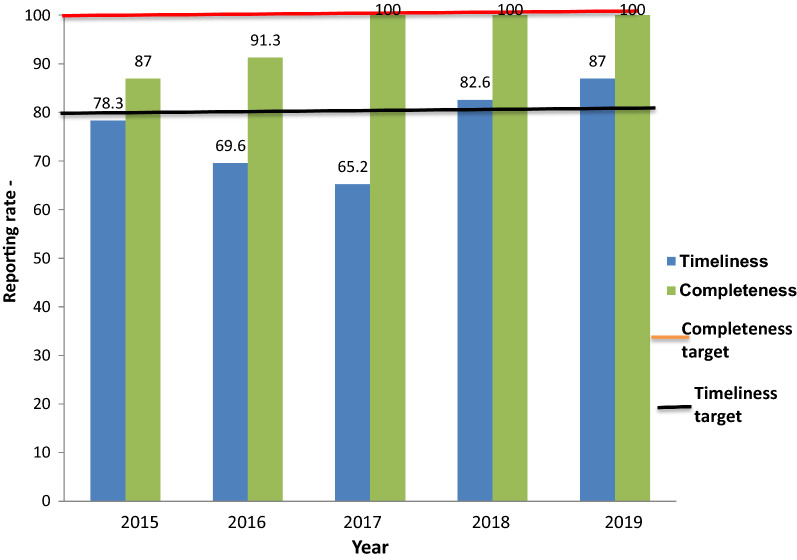
Annual reporting rates for timeliness and completeness in Benue State, 2015–2019

The surveillance system’s timeliness was 76.5% on average. The highest percentage was in 2019 (87.0%), while the lowest was in 2017 (65.2%). The annual target for timely reporting is 80.0 percent, and 100% for reporting rate (Fig. 5).

#### Stability

The surveillance system’s stability refers to its reliability (i.e., its capacity to collect, handle, and provide data without fail) and availability (its ability to be operational when needed). Data was collected via paper-based forms, computers and Android phones, but power disruptions posed a new challenge to the system’s stability. No RBM focal persons in the LGAs were transferred or replaced throughout the evaluation period. All the RBMs affirmed that there are dedicated staff for the management of data. Stock-outs of malaria commodities such as malaria RDT kits, LLITNs, and ACT, as well as decreased funding, were cited as limitations by 42 (91.3%) of the 46 RBM focal persons. Key stakeholders said the majority of the funding came from partners, with little to no support from the state or local governments (except for salaries) and the state conducts bimonthly integrated supportive supervision visits to health facilities, but these visits are conducted irregularly due to scarcity of funds.

## Discussion

The malaria surveillance system in Benue State is achieving most of its set objectives. According to stakeholders at the state level and RBMs at the local government levels, the system is simple and flexible. This could be due to the LGAs’ operators’ training and supervision, as well as the established clear structure and channel of communication that is fully understood by all. Other research in Nigeria and Bhutan [14–18] have found similar results, however studies in Yemen utilizing IDSR, and East Sumba District, India, contrast this finding [19, 20]. As modifications in the national diagnosis and treatment guidelines occurred, the system adapted well. Other researchers in Nigeria have shown similar results [14–17].

Over the years, the system has proven to be effective in detecting malaria cases. This is similar to what has been reported in Kano, Ebonyi, and Kaduna [14, 15, 17]. Malaria cases were more common from April through September each year during the evaluation period, with peaks in June and July. This is the rainy season, which creates an ideal setting for the Anopheline mosquitoes that transmit malaria to breed. This is similar to the research done in Ebonyi State, Nigeria [15].

The Malaria Surveillance System in Benue does not reflect all health facilities in the state because data is only available from 888 primary and 24 secondary health institutions, with data available only from 25 of the 520 private health facilities and none from the 2 tertiary health facilities. This could be due to a lack of awareness of the malaria surveillance system’s existence, a lack of harmonized health management information system data capturing tools, and the surveillance system's operators' lack of involvement in the management of these private and tertiary health facilities in Benue State. This finding was consistent with earlier research [14–17, 20]. There is need to integrate the private health sector and the tertiary health to the malaria surveillance system so that the data generated could be more representative of the system.

The Benue malaria surveillance system met the target of 80% for timeliness of reporting in the last 2 years (2018 & 2019) and 100% for completeness of reporting in the last 3 years (2017–2019) of the evaluation. This finding is similar to the findings in a study in Ebonyi State Nigeria where timeliness of reporting was achieved [15], However this is in disagreement to findings in Kano and Kaduna States in Nigeria, where target of completeness was not achieved [14, 17]. Reporting accuracy and timeliness are essential for planning and making timely decisions. Delayed and inadequate reporting of a surveillance system's can make it difficult to assess disease and detect outbreaks [21].

There are certain limitations to the research. The malaria surveillance data in Benue State is only from public health institutions, therefore the findings may not be a real reflection of the state's malaria burden, which has implications for the elimination effort. Secondly, because the Nigerian national malaria diagnosis and treatment guidelines use RDT or microscopy for malaria diagnosis rather than screening tests, the predictive value and sensitivity could not be evaluated.

## Conclusion

Surveillance is a great tool towards achieving elimination of malaria in Benue state. The findings from the evaluation of the malaria surveillance system in Benue State revealed that the system was useful, simple, flexible and acceptable; however there is need to integrate tertiary and private health facilities into the system to improve representativeness, and maintain timeliness and completeness of reporting that was achieved in the last 2 years and last 3 years of the evaluation period, respectively. This is important for planning and implementing targeted interventions and measuring progress towards elimination. Findings from the evaluation have been shared with the Benue state malaria elimination programme key stakeholders. The state malaria key stakeholders should ensure that tertiary and private health facilities in the state are part of the malaria surveillance system to enhance the utilization of reports and representativeness and to provide regular capacity building and ensure regular supportive supervision for RBM focal persons at the LGAs to enhance staff retention and improve data quality.

## Data Availability

The data generated for this research is available from the corresponding author on reasonable request.

## Abbreviations

ACT: Artemisinin-based combination therapy
BSMEP: Benue state malaria elimination programme
CDC: United states centers for disease control and prevention
DHIS: District health information system
DSNO: Disease surveillance and notification officer
GTS: Global technical strategy
KII: Key informant interview
LGA: Local government area
M&E: Monitoring and evaluation
NMEP: National malaria elimination programme
RDT: Rapid diagnostic test
RBM: Roll back malaria
WHO: World health organization
